# Biosensing Technology to Track Adherence: A Literature Review

**DOI:** 10.3390/healthcare9101339

**Published:** 2021-10-09

**Authors:** Cody K. Dukes, Elizabeth A. Sheaffer

**Affiliations:** 1 Alumnus, McWhorter School of Pharmacy, Samford University, Birmingham, AL 35229, USA; cdukes@samford.edu; 2 Department of Pharmaceutical, Social, and Administrative Sciences, McWhorter School of Pharmacy, Samford University, Birmingham, AL 35229, USA

**Keywords:** biosensing technology, digital medicine system, medication adherence, medication event monitoring system, nonadherence, pharmacy

## Abstract

Tracking adherence can be a useful means of identifying opportunities to provide educational intervention to nonadherent patients. The aim of this study was to evaluate the ability of biosensing technology to track medication adherence. Searches of PubMed and Ovid IPA were conducted. The criteria for inclusion were studies that tracked and reported ingestion events. Studies that did not track ingestion events were excluded from this review. Titles and abstracts were assessed for relevance, and full-text reviews were performed on all potentially relevant studies. References from the studies retrieved from the literature searches were assessed for additional applicable articles. Overall, ingestion events were detected 91.3% of the time, with many of the failed detections being related to patients not using or inappropriately using the system. In the studies that looked at the latency time, the overall mean time to detection by the wearable sensor was between 1.1 and 5.1 min. With medication nonadherence being a persistent problem in healthcare, biosensing technology presents an innovative approach to tracking adherence. The technology has been shown to be accurate in its ability to track actual medication use in patients. It has also been shown to detect ingestions with a minimal delay after administration. Accessibility may be an issue with this technology in the future, and further studies may be necessary to access the viability of biosensing technology.

## 1. Introduction

Medication adherence is of critical importance in today’s healthcare system. Adherence can be described simply as the extent to which a patient follows through in sticking to a planned regimen for his/her treatment from a health care provider [1]. This follow-through can often be the linchpin in a patient’s health. It is generally accepted that an adherence rate of at least 80% is required to achieve optimal therapeutic outcomes [2]. However, medication nonadherence is very prevalent in the United States. It is estimated that nonadherence accounts for up to 50% of failures in treatment, about 125,000 deaths, and around 25% of hospitalizations each year [2].

The responsibility of adherence is not solely a patient-based issue. This problem falls on the shoulders of patients and practitioners alike. Patients are ultimately responsible (in most cases) for the administration of their medication, but there are other steps in the healthcare process that are important in reducing nonadherence. Doctors can explain the necessity of consistently using a medication regimen when prescribing to patients. Nurses can emphasize adherence and ensure patient understanding during transitions of care and discharges. Pharmacists can educate patients on how the medications work, why they are being used, and how often they are to use them. A breakdown at any of these stages or others in the healthcare system can be the cause of medication nonadherence.

New technologies are frequently being implemented to try to curtail this problem. The subjective and often inaccurate feedback associated with pill counts and self-reports has not been very successful in achieving adherence in patients. Healthcare has begun to look at utilizing technology as a path to possible solutions. The advent of mobile technology has allowed for a variety of ways to help with medication adherence. This makes sense as most adult Americans now own a cell phone. Mobile devices have several functions that lend themselves to healthcare, such as phone calls, text messaging, and mobile applications. Due to this functionality, we have seen the utilization of these functions in the effort to increase medication adherence. Some institutions practice automated calling to serve as reminders for patients to take their medications or show up for appointments. Others have implemented automated text messaging to provide a similar reminder to patients. Various applications have been created to help with adherence. Some practitioners will suggest these applications to their patients, or the patients will find one that they find convenient of their own volition. Many of these have shown effective results in previous studies, but medication nonadherence is still an issue, and the healthcare system still seeks to find ways to improve adherence.

Numerous other methods currently exist to directly and/or objectively assess adherence, including pill counts, Medication Event Monitoring System (MEMS) bottle caps, pharmacy refill records, and biological assays from bodily fluids [3]. However, they all have limitations, and none provide an actual measure of medication ingestion. Therefore, the ability to precisely and objectively assess medication adherence in patients is a significant unmet need [3].

A newer technological advancement that may have a profound effect on increasing (or at least monitoring) adherence is biosensing technology. This technology works by having patients consume medication in a special formulation that allows it to be tracked outside of the body. This technology also includes the use of mobile technology and may be of great use in nonadherent patients. This technology not only allows the patients to track their own administration habits, but it also allows prescribers to track the patient’s adherence to medication regimens in order to make changes to the regimen and/or counsel patients on the need to be adherent. This technology has the potential to eliminate the guesswork associated with whether a patient is taking his or her medications.

Digital medicine systems (DMSs) are a newly designed technology that has been developed for the purpose of tracking the ingestion of medication. They provide a more accurate and objective measure for tracking adherence than a patient’s self-reporting or pill counting. “DMSs combine the proven safety and efficacy of orally administered medications with the ability to electronically confirm medication ingestion and send feedback to the patient, health care provider, and elected others such as caregivers or family members” [4].

**Figure 1:**
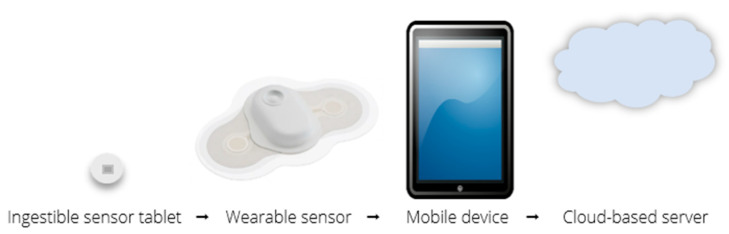
Digital medicine system data flow.

The digital medicine system consists of three integrated components: an ingestible sensor in tablet form, a wearable sensor, and a mobile/cloud-based computing system [4]. See Figure 1 for a visualization of the data flow. The system works by a dose of medication preformulate with the ingestible sensor being placed in a tablet. Once the tablet is ingested and activated in the stomach, the data is transmitted to the wearable sensor. The wearable sensor relays the ingestion to the application on the patient’s mobile device, which records the ingestion event on the cloud server. This allows for the information to be accessed by providers.

There are important aspects of this technology that must exist for it to serve as a viable strategy to affect adherence rates:

- Accuracy: The system must be able to accurately track ingestion events (adherence).
- Tablet to Sensor Latency: The system must be able to relay a tablet ingestion to the wearable system in a reasonable amount of time.
- Sensor to Mobile Application/Cloud Server Latency: The system must be able to communicate the data received by the wearable sensor to the mobile device or cloud-based server in a reasonable amount of time.

For a digital medicine system to serve as a worthwhile response to nonadherence, it must ensure that all three of the above-mentioned requirements are met, or it would not warrant the trouble of using such a technology as it would not be cost-effective [4].

The objective of this review is to assess the ability of the DMS to track adherence by examining available data pertaining to its capability to track ingestion events. Like many other new technologies, the DMS comes with a steep price tag. A currently available DMS, the Abilify Mycite^®^, costs approximately $2000 for a month’s supply.

## 2. Methods

A systematic literature review and analysis was performed for this study. To identify relevant publications, PubMed and Ovid International Pharmaceutical Abstracts (IPA) were searched for all articles relevant to the study regardless of the publication date. First, a search of PubMed MeSH terms was conducted. The search terms were as follows: Medication Adherence AND (Biosensing Techniques OR Radio Waves OR Radio Frequency Identification Device). Then, a free-text search of PubMed was conducted. The search terms were as follows: Medication adherence AND (Biosensing techniques OR Radio waves OR Radio frequency identification device). Then, the same free-text search was conducted on the Ovid IPA database. The search terms were as follows: Medication adherence AND (Biosensing techniques OR Radio waves OR Radio frequency identification device). Titles and abstracts were assessed for relevance, and full-text reviews were performed on all potentially relevant studies. References from the studies retrieved from the literature searches were assessed for additional applicable articles.

Studies that tracked and reported ingestions using a DMS were included. Studies that did not track and report ingestions of a DMS were not included. As there is not much literature that exists on the subject, studies meeting inclusion criteria were identified and included. Two studies that returned from the search were excluded from this review as they did not track ingestions of a DMS. The extracted data included the clinical setting, purpose, methods, population, accuracy of the digital medicine system in tracking ingestion events, and the latency of the data transmission.

## 3. Results

The search of the literature produced four total studies that met the criteria for inclusion and two that did not meet the inclusion criteria. In the four studies that were included, biosensing technology was found to capture 86.3% of ingestions events. When accounting for a transmission issue in one of the studies, 91.3% of tablets formulated with the digital medicine system were captured in the studies. See Table 1 for the study comparisons, including the purpose, methods, population, accuracy, and latency.

Ten participants were included in a study conducted at an emergency department where oxycodone was the medication within the digital medicine system. A pill count was used to verify the fidelity of the system. Of the 110 pills that were taken, 96 ingestion events were recorded by the system (87.3% accuracy) [5]. The 14 missed events were accounted for by two participants, both of whom refused to use the system [5]. Therefore, these 14 missed events were considered as nonadherence by two of the ten participants. It can be inferred that the system would have otherwise detected 100% of the ingestion events. In this study, the system received a 90% acceptance rate from participants [5].

A similar study was conducted in an emergency room that also used oxycodone as the medication within the digital medicine system. Sixteen individuals consented to participate in this study, but only fifteen completed the study. A pill count was also used to verify the fidelity of the system in this study. The digital medicine system recorded 112 ingestion events, while the pill counts suggested 134 total pills ingested (83.6% accuracy) [6]. Similar to the other study, all missed doses were accounted for by two participants who failed to properly use the system [6]. It can similarly be inferred that the detection rate would have been 100% otherwise.

Two sub-studies were conducted as part of a study examining the aripiprazole digital medicine system. These studies not only looked at accuracy but also latency. In the first sub-study, 30 participants were enrolled and completed the study. Participants were taking one of the digital medicine system tablets at four time points. The tablet at the first time point contained aripiprazole, and the tablets at the other three time points contained a placebo. The overall accuracy (overall ingestion detections at the four time points) was 78.3% (94/120 events detected) [4]. However, a post hoc analysis of the information transmission at each stage showed that the wearable sensor had a much higher rate of detection at 98.3% (118/120 events detected) [4]. This implies that somewhere between the transmission from the wearable sensor to the mobile application to the cloud-based server, there was a breakdown that caused the ingestion event not to be recorded at every step. It should be noted that this breakdown was the product of two factors: (1) an early version of the application used in this sub-study did not properly check for a complete data transfer from the wearable sensor to the application, and (2) the protocol for this sub-study did not emphasize to patients the option of a forced data upload from the wearable sensor before the removal of the sensor after each ingestion event [4].

In the other sub-study, 29 individuals enrolled in and completed the study. In this study, the results from the previous sub-study were used to update the software and improve the outcomes. Participants were similarly using the digital medicine systems at four time points. The wearable sensor detected ingestion events between 93.1% and 100% for all four time points [4]. The overall accuracy of detected ingestions was 96.6% (112/116 events detected) [4]. This was consistent with the accuracy reported in the previous study.

The mean latency time from the actual ingestion events to signal detection by the wearable sensor at the four time points was between 1.1 and 1.3 min. Seventy-seven point six percent (77.6%) of ingestions (90/116 ingestion events) were detected between 1 and 3 min, with 16.4% (19/116) of ingestions detected in less than 1 min [4]. The mean latency time from the wearable sensor detection of an ingestion event to the cloud-based server detection of that same ingestion event at the four time points was between 6.2 and 10.3 min [4]. “50% of transmissions from the wearable sensor to the server were completed in less than 2 min, and approximately 90% (105/116) of all ingestion events were registered by the mobile application within 30 min from ingestion.” [4]. In both sub-studies, the mean times of latency between the sensor ingestion and detection by the wearable sensor were 1.1 and 5.1 min for sensors in the placebo and aripiprazole tablets, respectively [3].

Overall, 86.3% (414/418) of ingestion events were detected in all studies. This rate is increased to 91.3% if adjusted for the detections with incomplete transmissions recorded in the first sub-study of the aripiprazole trials.

## 4. Discussion

The digital medicine system is a new and exciting technology that might serve as a method to help improve therapeutic outcomes by improving adherence. Biosensing technology gives both prescribers and patients the ability to accurately determine the level of adherence to a specific medication regimen. It has shown accuracy in detecting ingestion events in two different drug classes where adherences to a medication regimen are pivotal in order to reach positive therapeutic outcomes. In opioids, where monitoring a patient’s medication usage could be vital to pain control and avoiding overuse, it has proven to be a viable option in accurately detecting adherence. Likewise, in the antipsychotic drug class where a near-perfect adherence is necessary in order to remain effectively treated but where 40–50% of patients being treated for serious mental illnesses are estimated to be nonadherent, the digital medicine system has been demonstrated to be an option for providers to be able to ensure that their patients are sticking to their treatment plans [4].

It is suggested that an adherence rate of at least 80% is generally necessary to reach desired therapeutic outcomes [2]. In all the studies reviewed, more than 80% of ingestions were detected. Many of the failed detections were a result of a user error, and it could be inferred that if not for the user error the digital medicine system would have detected more than 90% of all ingestions in each of the studies reviewed. Despite this fact, ingestions were detected at an overall rate of 91.3%. Therefore, the DMS consistently demonstrated the ability to track adherence rates that were congruent with positive outcomes in therapy.

In the two sub-studies examining the aripiprazole DMS, the mean latency times were reported at 1.1 and 5.1 min [3]. This demonstrated that biosensing technology not only provided a consistent accuracy needed to track adherence but also provided a short latency time that allowed the technology to be viable as a means of tracking adherence. However, further studies evaluating the latency times of digital medication systems are necessary in the future in order to prove consistently short latency times with the use of digital medication systems.

While the accuracy and latency of the biosensing technology would make a digital medicine system favorable (if not preferable) in the setting of treating patients with nonadherence issues or prescribing drugs that require consistent administration, there still remains the issue of cost. Because this is a fairly new technology, it is not marketed for many drugs, and it is likely to be expensive. The Abilify Mycite^®^ (aripiprazole DMS) tablet is approximately $66 per DMS (about $2000 per month). It is unlikely that insurances would cover it and even more unlikely that patients would be willing to pay for it out-of-pocket. Thus, while this technology has brought medication monitoring a long way in its ability to accurately and promptly detect adherence, it still has a long way to go before the population at large will have access to it.

In a recent study [7], the ethical nature of the DMS has been questioned and is still a concern that should be considered before making a tablet containing a sensor a common practice in healthcare. Another consideration is whether or not a technology that relies on a patch is optimal for tracking adherence. Patches tend toward user errors and therefore could render the DMS less reliable.

Another study [8] calls into question whether or not enough rigorous evidence is available to justify the use of the DMS. The authors’ primary concern is the use of this new delivery system as a means to repackage and extend the life cycle of a drug nearing the end of its patent-protected exclusivity without any noticeable improvement in outcomes.

The studies included in this review were appropriate as they all used a DMS and reported on actual ingestions. This limitation of this review is the apparent lack of available studies conducted using the DMS technology, which may not provide a great deal of evidence for the mainstream applicability of this technology.

## 5. Conclusions

The advent of the digital medication system utilizing biosensing technology can have a significant impact on monitoring and tracking adherence, especially in certain at-risk medication classes. It has shown both a good accuracy and acceptable latency in the detection of actual ingestions in patients administering medications that require close monitoring (opioids and antipsychotics). If properly used, this technology can be helpful in the monitoring of other high-risk medication classes (anticoagulants, antiretrovirals, etc.). Limited data exist on latency times, and while more research needs to be completed relating to the overall viability of this technology, it currently appears to be a promising advancement in the healthcare system to help tackle the issue of medication nonadherence.

The lack of existing data on the ability of the DMS to track adherence is likely a sign of the need for more studies before the technology is used more commonly. This lack of data could also be a sign of a couple of obstacles to the commonplace use of the technology: the cost and necessity (cost vs. benefit). Increasing cost to the healthcare system might not be worth the ability to more closely monitor adherence when there is always the option to count pills as was done in the past.

## Figures and Tables

**Table 1:** Biosensing Study Comparisons.

Study	Digital Pills to Measure Opioid Ingestion Patterns in Emergency Dept. Patients with Acute Fracture Pain: A Pilot Study [6]	Oxycodone Ingestion Patterns in Acute Fracture Pain with Digital Pills [5]	Developing a Digital Medicine System in Psychiatry: Ingestion Detection Rate and Latency Period, Substudy A [3]	Developing a Digital Medicine System in Psychiatry: Ingestion Detection Rate and Latency Period, Substudy B [4]
**Purpose**	Determine feasibility of a digital medication system (DMS); patients needing as-needed pain medication after fractures	Measure as-needed pain medication utilization after acute fractures	Measure accuracy and latency of detections by a DMS	Measure accuracy and latency of detections by a DMS
**Methods**	Patients received a DMS containing oxycodone; use was tracked for one week	Patients received a DMS containing oxycodone; use was tracked for one week	Patients received a DMS containing aripiprazole/placebo; detection and latency were measured	Patients received a DMS containing aripiprazole/placebo; detection and latency were measured
**Population**	10 participants	15 participants	30 participants	29 participants
**Accuracy**	96/110 (87.3%) ingestion events detected	112/134 (83.6%) ingestion events detected	94/120 (78.3%) ingestion events detected	112/116 (96.6%) ingestion events detected
**Latency**	Not tracked	Not tracked	Mean latency time of 1.1 (placebo) minutes and 5.1 (aripiprazole) minutes by wearable sensor [118/120 ingestions detected by wearable sensor]	

## Data Availability

Not applicable.
